# Prognostic value of sirtuin family members and experimental verification identify SIRT5 as diagnostic biomarkers in clear cell renal cell carcinoma

**DOI:** 10.7717/peerj.15154

**Published:** 2023-04-19

**Authors:** Lu-Shan Peng, Sai-Li Duan, Run-Qi Li, Zi-Yuan Bai, Chun-Lin Ou, Jun-Pu Wang

**Affiliations:** 1 Department of Pathology, Xiangya Hospital, Changsha, Hunan, China; 2 Department of General Surgery, Xiangya Hospital, Changsha, Hunan, China; 3 Department of Pathology, School of Basic Medicine, Central South University, Changsha, Hunan, China; 4 Department of Pathology, First Affiliated Hospital of Kunming Medical University, Kunming, Yunnan, China; 5 National Clinical Research Center for Geriatric Disorders, Xiangya Hospital, Changsha, Hunan, China; 6 Key Laboratory of Hunan Province in Neurodegenerative Disorders, Xiangya Hospital, Changsha, Hunan, China

**Keywords:** Bioinformatics, Immune cell infiltration, Renal cell clear cell carcinoma, Prognosis, Sirtuin

## Abstract

**Background:**

The sirtuins (SIRTs) family is a nicotinamide adenine dinucleotide (NAD+) family of dependent deacetylases, which includes SIRT1-7. This family is related to the development and progression of various tumors. However, a comprehensive analysis of the role of SIRTs in clear cell renal cell carcinoma (ccRCC) is still lacking, and there are few reports on the inhibitory role of SIRT5 in ccRCC.

**Methods:**

We used immunohistochemical analysis, and several bioinformatic databases to perform an integrated analysis of the expression and prognostic value of SIRT5 and other SIRT family members in ccRCC along with the associated immune cell infiltration. These databases include TIMER, THPA, cell culture, UALCAN, cBioPortal, WebGestalt, Metascape, DiseaseMeth, STRING database, and Cytoscape.

**Results:**

The protein expression of SIRT1, 2, 3, 6, and 7 were upregulated in ccRCC for the Human Protein Atlas database, whereas the expression of SIRT4 and SIRT5 was decreased. The expression based on tumor stage, and grade followed a similar trend. Kaplan–Meier analysis showed that high SIRT4 and SIRT5 expression was positively related to better overall survival (OS), whereas SIRT6 and SIRT7 expression was positively related to worse OS. Further, high SIRT3 expression was related to worse relapse-free survival (RFS), whereas high SIRT5 expression was related to better RFS. To explore the mechanism underlying the function of SIRTs in ccRCC, we also used several databases to perform the functional enrichment analysis and explore the relationship between infiltrating immune cells and seven SIRT family members in ccRCC. The results showed that several SIRT family members, and particularly SIRT5, are correlated with the infiltration of some important immune cells. The protein expression of SIRT5 was significantly lower in tumor tissue compared to normal tissue and was negatively related to the age of the patient ccRCC individual tumor stages, and grades. In human ccRCC samples, strong IHC staining expression of SIRT5 was displayed in adjacent normal tissue than in tumor tissues.

**Conclusion:**

SIRT5 may be a prognostic marker and a novel strategy for the treatment of ccRCC.

## Introduction

Clear cell renal cell carcinoma (ccRCC) is the most common tumor of the urinary system, accounting for approximately 85–90% of cases (Moch et al., 2016). Moreover, ccRCC is the most common form of kidney cancer, which is an aggressive cancer associated with high mortality (Motzer, Bacik & Mazumdar, 2004) and poor response to treatment (Chen et al., 2022). More than 10,000 deaths were attributed to ccRCC in the US in 2021 only (Siegel et al., 2021). Study showed that over 30% ccRCC patients were found distant metastases when diagnosed, and the 5-year survival rate of those patients is only 8% (Marona et al., 2017). To improve the long-term survival rate of patients with ccRCC, early detection and diagnosis is necessary. Consequently, there is an urgent need to explore novel biomarkers and therapeutic targets for the diagnosis and treatment of ccRCC. Viewed from a pathological point, the transparent morphology of ccRCC under the microscope correlates with the metabolites produced by its cancer cells, so we believe that finding a metabolism-related biomarker may provide an important contribution to the early diagnosis of ccRCC.

The sirtuin (SIRT) family is a nicotinamide adenine dinucleotide (NAD^+^) family of dependent deacetylases (Zhao et al., 2022b), which are related to the development and progression of various tumors and function by regulating molecular signaling (Ezhilarasan et al., 2022). There are seven members of this family, including SIRT1, SIRT2, SIRT3, SIRT4, SIRT5, SIRT6, and SIRT7. Some of them can promote the development and progression of different tumors. For example, the upregulation of SIRT1 expression can promote non-small cell lung cancer metastasis by promoting EMT. SIRT2 was found to promote cell stemness while repressing chemosensitivity in endometrial cancer (Zhao et al., 2022a), whereas others are proved to be tumor suppressors. Huang et al. (2021) speculated that SIRT4 could induce cellular senescence and cell apoptosis in hepatocellular carcinoma (HCC) cells, which could be helpful for future anticancer strategies. One study showed that SIRT5 was found to suppress the development of HCC (Sun et al., 2022). In ccRCC, SIRT family members are thought to regulate tumorigenesis, for example, SIRT5 downregulation was thought to promote metabolic reprogramming of ccRCC, the expression of SIRT5 was found to be decreased, and its downregulation can induce tumorigenesis and progression by accelerating the Warburg effect through PDHA1 hyper-succinylation, indicating that this marker might be a possible ccRCC suppressor (Yihan et al., 2021). Although some SIRT members were proven to be important for the development and progression of ccRCC, for example, Tan et al. (2020) made preliminary analysis of sirtuins in ccRCC, there has still been no systematic and comprehensive study on the role of the SIRT family in ccRCC combined experiments, and few reports on the inhibitory role of SIRT5 in ccRCC and its influence toward the prognosis of patients with ccRCC.

In this study, we used immunohistochemical analysis, and several bioinformatic databases, such as gene expression profiling interactive analysis (GEPIA), UALCAN, cBioPortal, WebGestalt, and Metascape, to analyze the function of SIRT5 and other six SIRT family members in ccRCC, which could be helpful in finding new strategies for ccRCC treatment.

## Methods

### Timer

TIMER is a comprehensive resource for systematical analysis of immune infiltrates across diverse cancer types (Li et al., 2020). We used this database to get the scatterplots that show the correlation of SIRTs with six types of infiltrated immune cells and their signature markers.

### The human protein atlas

The Human Protein Atlas (HPA) is a useful database based on proteomic, transcriptomic and systems biology data, it can map tissues, cells, and organs (Thul & Lindskog, 2018). Not only tumor tissues, but also the protein expression of normal tissues is included. The survival curves of tumor patients can also be viewed. We used this database to obtain the immunohistochemistry images of SIRTs in ccRCC and in normal tissues.

### Immunohistochemistry

We purchased a tissue microarray containing slides of 90 ccRCC tumor tissues and adjacent normal kidney tissue from Outdo Biotech (HKidE180Su03; Outdo Biotech, Shanghai, China) with patients’ clinical records, including histopathological diagnoses and prognostic information. We used SIRT5 (Proteintech, #15122–1-AP, 1:200) as primary antibodies, and incubated with it overnight at 4 °C. The sections were then incubated with the secondary antibodies and DAB regents for staining (Zhong Shan Golden Bridge Biotechnology, Beijing, China). The results of IHC were scored by two independent pathologists respectively according to both staining intensity that contain negative, weak, moderate and strong, corresponding to 0, 1, 2, and 3, respectively and percentage of immunoreactive tumor cells (<5% = 0, 5–25% = 1, 26–50% =2, 51–75% = 3, >75% =4). The final expression level score was obtained by multiplying the above two scores (ranging from 0 to 12). The samples with score <3 were considered as negative expression, and ≥ 3 were considered as positive expression.

### Data analysis

#### Date description

In this article, we used IHC, statistical analysis based on IHC scores, and several bioinformatic databases to evaluated the expression and prognostic value of SIRT5 and other SIRT family members in ccRCC along with the associated immune cell infiltration. We selected the data of 90 patients’ life cycles (weeks), sex, age, pathological grade, tumor size, TNM grade and the indicators beside cancer. During the data preprocessing, the pathological grade I-II and III-IV were classified as category data; the tumor size was marked as category data with the maximum diameter of 6.0 cm as the boundary; the T grade was marked with T1-T2 and T3 as category data; the N grade was marked with N0 and N1-N2 as category data; and the data of the indicators beside the cancer were given by experts. If it was less than or equal to 3, it was marked as negative, otherwise it was marked as positive.

#### Statistical analysis

We used chi-square testing and Fisher’s exact test to achieve univariate analysis of paracarcinoma and other clinical indicators. The corresponding multi-factor correlation analysis is implemented using logistic regression. Combined with case survival data, univariate analysis of variance (ANOVA) was used by Kaplan–Meier to plot survival curves and use a Log-rank test to compare groups with significantly isolated survival curves. Multivariate survival analysis is given by Cox regression analysis. The methods and results adopted by the Institute rely on Python third-party packages lifelines, scipy.stats, and statsmodels implementations. A *p*-value of 0.05 or less than or equal to the test result is considered statistically significant.

#### UALCAU

An effective online cancer data analysis website, mainly based on the relevant cancer data in the TCGA database (Chandrashekar et al., 2017). It can perform biomarker identification, expression profiling, survival analysis on related genes. We use this database to assess the seven SIRTs family members mRNA expression, and their expression based on the tumor stage, grade.

#### cBioPortal

Providing visualization tools to study and analyze cancer genetic data, cBioPortal helps molecular data from cancer histology and cytology studies to recognize and understand genetics, epigenetics, gene expression and proteomics (Unberath et al., 2019). By using the cBioportal database, we explored the coexpression profiles of the SIRTs family in ccRCC.

#### WebGestalt

WebGestalt is an online website focused on enrichment analysis, which covers a comprehensive functional annotation database and can be used to do a variety of enrichment analysis algorithms (Liao et al., 2019). By using this database, we analyzed the GO functional enrichment and the KEGG pathway of the SIRT-related co-expressed molecules.

#### STRING and Cytoscape

STRING is an online database for searching protein-protein interaction information (Szklarczyk et al., 2019). Cytoscape is a software that graphically displays networks for analysis and editing. It supports a variety of network description formats, as well as tab-delimited text files or Microsoft Excel files as input, or to build networks directly using the software’s own editor module. STRING and Cytoscape are used together to construct the PPIs network.

#### Metascape

Metascape database is a powerful gene function annotation analysis tool that integrates multiple authoritative functional databases such as GO and KEGG (Zhou et al., 2019). This database was used for GO term enrichment analysis.

#### DiseaseMeth

DiseaseMeth is a database focused on abnormal methylation of human diseases, including a wide range of cancers, as well as datasets on neurodevelopmental and degenerative diseases, and autoimmune diseases (Xing et al., 2022). We use this database to explore the methylation levels of the seven SIRT family members.

## Results

### Abnormally-increased expression of SIRT family members in patients with ccRCC

**Figure 1:**
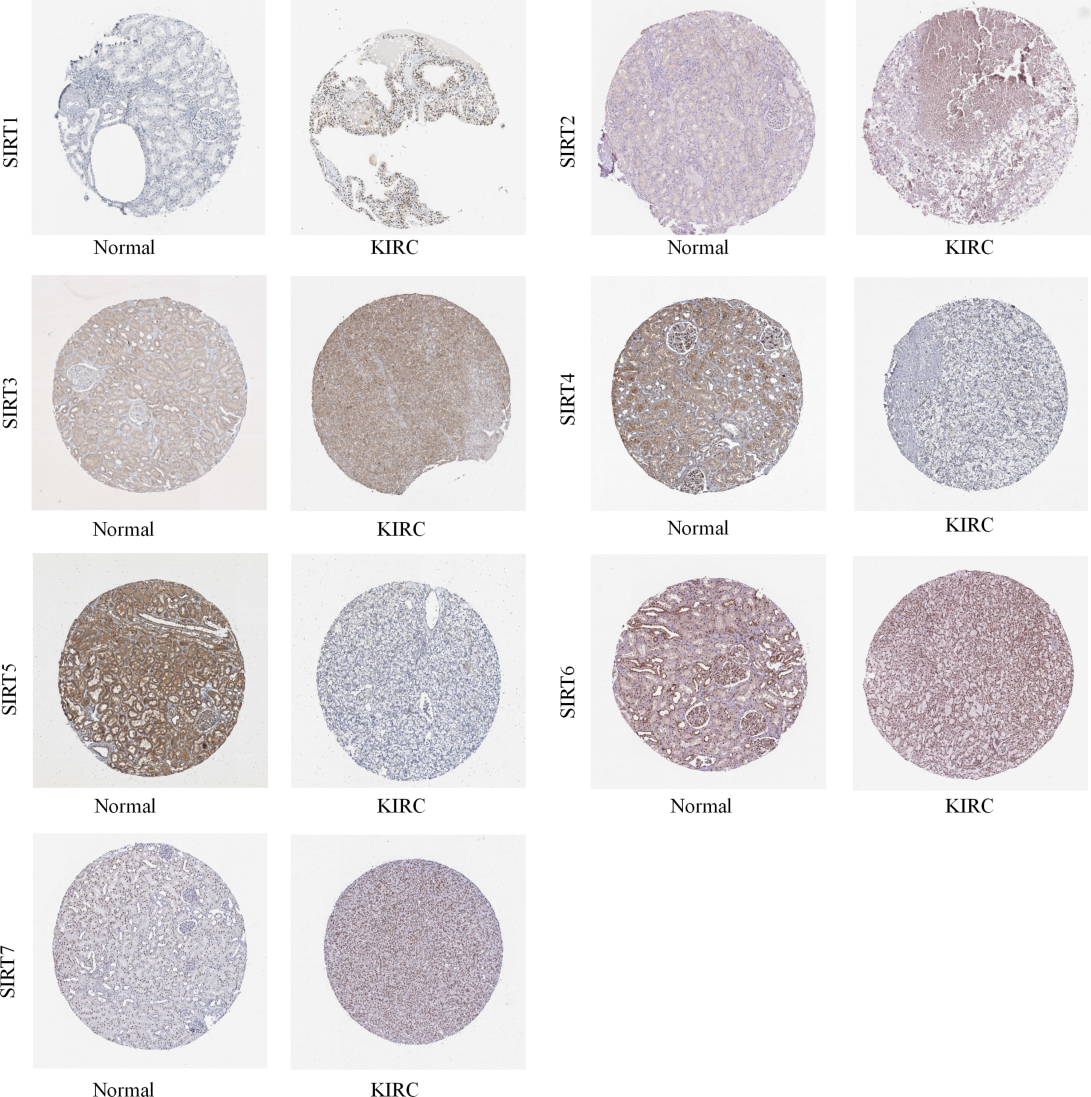
SIRTs protein expression in ccRCC and normal tissue. The THPA database was used to collect IHC images of SIRTs from ccRCC tissues and normal kidney tissues, representing the SIRTs protein expression levels.

Then, we used the Human Protein Atlas database to evaluate the protein expression levels of the seven SIRT family members in normal and ccRCC tissues. As shown in Fig. 1, the immunohistochemistry (IHC) staining data from the THPA database showed increased protein expression levels of SIRT1, SIRT2, SIRT3, SIRT6, and SIRT7, whereas the expression of SIRT4 and SIRT5 was significantly lower (particularly SIRT5), in ccRCC tissues. Thus, we speculated that SIRT5 may be an important tumor suppressor gene.

### Clinical-pathological characterizations of SIRT5 and other SIRT family members with ccRCC

Next, we want to explore the influence of SIRT5 and other SIRT family members on the clinical–pathological characteristics of patients with ccRCC.

**Figure 2:**
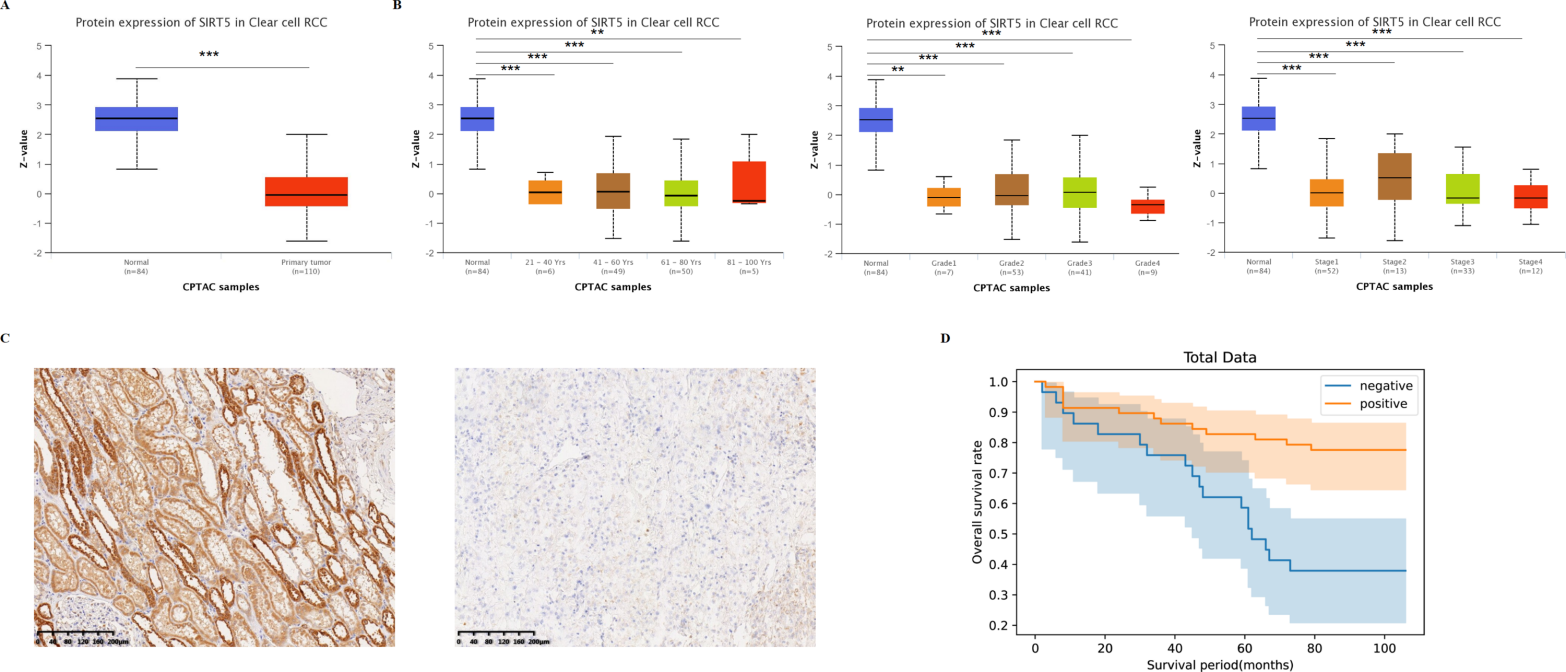
SIRT5-associated clinical pathology characteristics of patients with ccRCC. The UALCAN database was used to analyze the associations between SIRT protein expression levels (A) and its relationship with patients’ age, individual cancer stages as well as tumor grade of ccRCC (B). IHC staining (C) analysis showed the protein expression of SIRT5 in tumor compared with adjacent normal tissue. (D) Overall survival curves were plotted by using the Kaplan–Meier method. *: *p* < 0.05, **: *p* < 0.01, ***: *p* < 0.001.

To understand disease prognosis and outcomes, the UALCAN database was used to investigate the protein expression of SIRT5 (Fig. 2A) and the relationship between it and patients’ age, ccRCC individual tumor stages, and grades (Fig. 2B). The results showed that the protein expression of SIRT5 was significantly lower in tumor tissue compared to normal tissue and was negatively related to the age of the patient ccRCC individual tumor stages, and grades. To further verify it, we performed IHC on a tissue microarray containing slides with patients’ clinical records (including histopathological diagnoses and prognostic information). And in 90 human ccRCC sample, strong IHC staining expression of SIRT5 was displayed in adjacent normal tissue than in tumor tissues (Fig. 2C showed the representative images). Based on the IHC staining results, we made univariate (Table 1) and multivariate (Table 2) analysis results between SIRT5 protein expression and clinicopathological features. By using univariate analysis, aberrant expression of SIRT5 in ccRCC was closely related with pathological grading and cancer size with significant statistically difference (*p* < 0.05), among them, the *p*-value of 0.0614 was greater than 0.05 under the chi-square test for pathological grading index, but this study was based on a small sample, so the *p*-value of 0.0487 from Fisher’s exact test was adopted. By using Logistic regression test, we found that age (*p* < 0.005), pathological grading (*p* < 0.005), T stage (*p* < 0.005) and SIRT5 expression (*p* < 0.05) were four independent risk factors (visualization chart is shown in Fig. 2D). Consequently, we believe that SIRT5 plays important role in tumor suppression, and closely related to better prognosis of patients with ccRCC.

**Figure 3:**
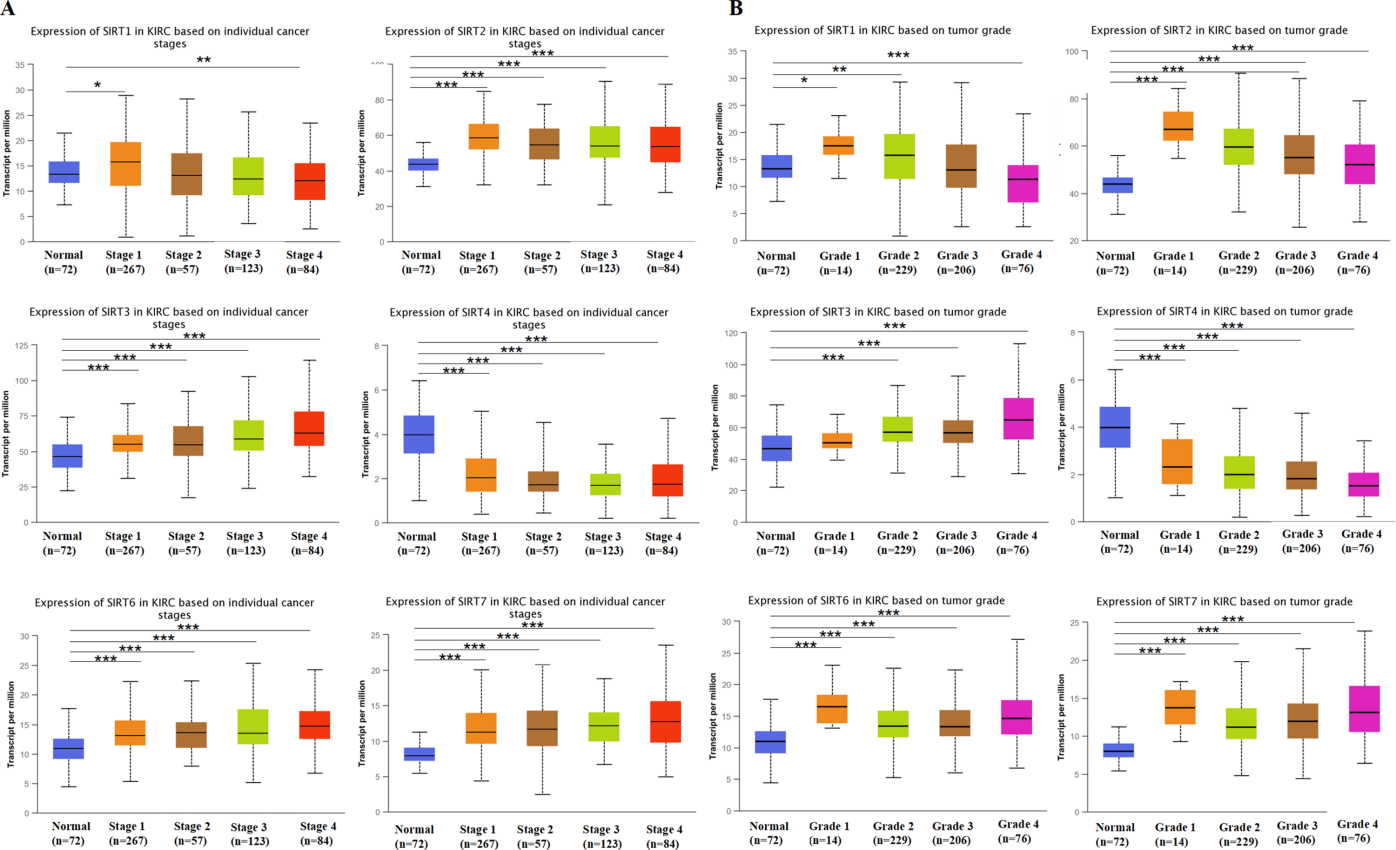
SIRT1, SIRT2, SIRT3, SIRT4, SIRT6, SIRT7-associated clinical pathology characteristics of patients with ccRCC. The UALCAN database was used to analyze the associations between SIRT transcript levels and individual cancer stages (A) as well as tumor grade (B) of ccRCC. *: *p* < 0.05, **: *p* < 0.01, ***: *p* < 0.001.

Further, we explored the function of SIRT1, SIRT2, SIRT3, SIRT4, SIRT6, and SIRT7 of patients with ccRCC. Studies have shown that the expression of SIRT2, SIRT3, SIRT6, and SIRT7 is increased in tumor-stage 1-4 subgroups, SIRT1 expression is increased in tumor-stage 1, and the expression of SIRT4 is decreased (Fig. 3). The expression of SIRTs with different tumor grades was the same. These results indicate that SIRT4 may play important roles in tumor suppression, whereas the other family members could be involved in driving the progression of ccRCC.

**Table 1:** Univariate analysis results between IHC staining results of SIRT5 and clinicopathological features.

	Negative (*n* = 39)	Positive (*n* = 51)	Total	*χ*^2^ test		Fisher’s exact test	
				*χ* ^2^	*p*-value	z	*p*-value
Gender				1.8379	0.1752	0.4755	0.1512
Male	16	44	60				
Female	13	17	30				
Age				2.2104	0.1371	0.4583	0.1113
≤60	13	39	52				
>60	16	22	38				
Pathological grading				3.4983	0.0614	0.3666	**0.0487**
I–II	16	47	63				
III–IV	13	14	27				
Cancer size				5.3484	**0.0207**	0.3043	**0.0163**
≤6.0cm	14	46	60				
>6.0cm	15	15	30				
T				0	1	0.9474	1
T1 and T2	27	57	84				
T3	2	4	6				
N				0.012	0.9129	1.9649	1
N0	28	57	85				
N1 and N2	1	4	5				
M				0	1	0.4667	0.5431
M0	28	60	88				
M1	1	1	2				

**Notes.**

Values with statistical significance are indicated in bold.

**Table 2:** Multivariate analysis results between IHC staining results of SIRT5 and clinicopathologic features.

	Coef	Exp (coef)	Se (coef)	Coef lower 95%	Coef upper 95%	Exp (coef) lower 95%	Exp (coef) upper 95%	Cmp to	z	*p*	−log2(p)
Sex	−0.52	0.6	0.45	−1.4	0.36	0.25	1.43	0	−1.16	0.25	2.02
Age	1.22	3.37	0.42	0.4	2.04	1.49	7.66	0	2.91	**<0.005**	8.09
Pathological grading	1.15	3.16	0.35	0.47	1.83	1.6	6.26	0	3.3	**<0.005**	10.02
Cancer size	0.48	1.62	0.4	−0.31	1.28	0.73	3.58	0	1.19	0.23	2.1
T stage	1.79	5.97	0.59	0.62	2.95	1.86	19.1	0	3.01	**<0.005**	8.58
N stage	0.56	1.75	0.41	−0.25	1.37	0.78	3.93	0	1.35	0.18	2.51
SIRT5 expression	−1.15	0.32	0.42	−1.98	−0.32	0.14	0.73	0	−2.71	**0.01**	7.21

**Notes.**

Values with statistical significance are indicated in bold.

### Association between prognosis and SIRTs in patients with ccRCC

**Figure 4:**
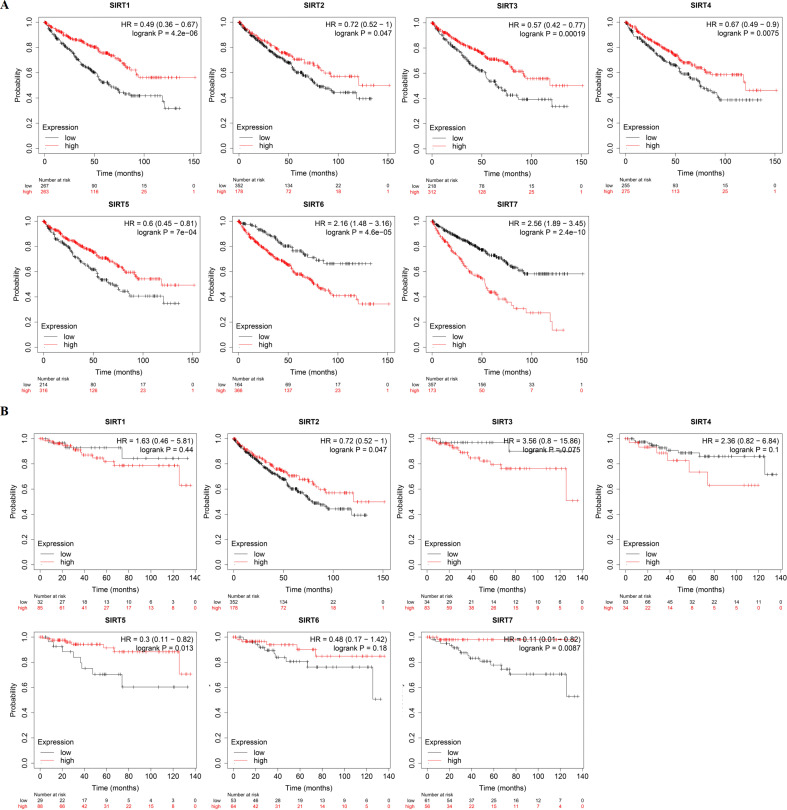
The prognostic value of SIRTs in patients with ccRCC. (A) The relationships between SIRTs transcript levels and OS of ccRCC patients. (B) The relationships between SIRTs transcript levels and RFS of ccRCC patients.

Next, we used the Kaplan–Meier database to generate overall survival (OS) curves and recurrence free survival (RFS) curves to explore the prognostic value of the SIRT family for patients with ccRCC (Figs. 4A and 4B). The OS and interactive correlations suggested that for patients with ccRCC, high SIRT4 and SIRT5 expression was significantly related to a better OS (*P* < 0.05). However, high SIRT6 and SIRT7 expression was shown to be related to worse OS (*P* < 0.05). The RFS results suggested that high SIRT3 expression was related to worse RFS (*P* < 0.05), whereas high SIRT5 levels were related to better RFS (*P* < 0.05). Therefore, SIRT4 and SIRT5 potentially inhibit the occurrence and development of ccRCC, whereas high expression of SIRT3, SIRT6, and SIRT7 might have an important tumor-promoting role.

### SIRT DNA methylation levels in patients with ccRCC

**Figure 5:**
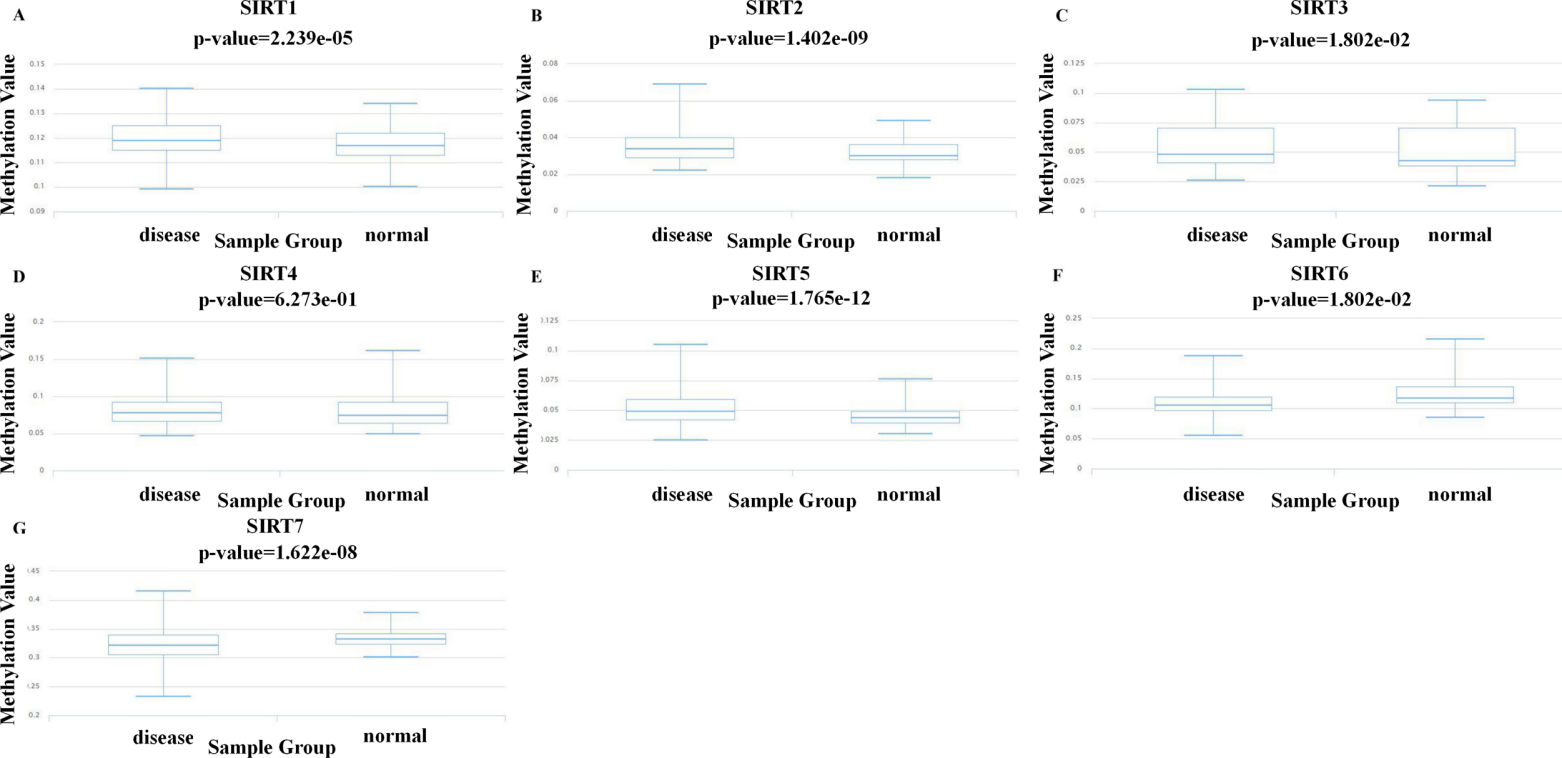
DNA methylation levels of seven SIRT family members in ccRCC. (A–F) We evaluated the DNA methylation values of SIRT1 (A) SIRT2 (B) SIRT3 (C) SIRT4 (D) SIRT5 (E) SIRT6 (F) and SIRT7 (G) between ccRCC tissues and normal kidney tissues by using DiseaseMeth.

Methylation is an important modification of proteins and nucleic acids, which regulates the expression and silencing of genes and is closely related to various diseases such as cancer. The study of methylation could be helpful for finding suitable drug targets for treatment. To explore new mechanisms to target for ccRCC treatment, we used the DiseaseMeth database to further investigate the levels of DNA methylation of SIRTs in patients with ccRCC. As shown in Fig. 5, the methylation of SIRT4 and SIRT5 was increased, whereas that of the SIRT6 and SIRT7 was decreased. Combined with SIRT expression (mRNA and protein) and the prognostic value of SIRTs (mentioned previously herein), we speculate that SIRT4 and SIRT5 might be possible tumor suppressor markers, whereas SIRT6 and SIRT7 could play an important role in promoting tumor development.

### Functional enrichment analysis of the SIRTs-associated coexpressed genes

**Figure 6:**
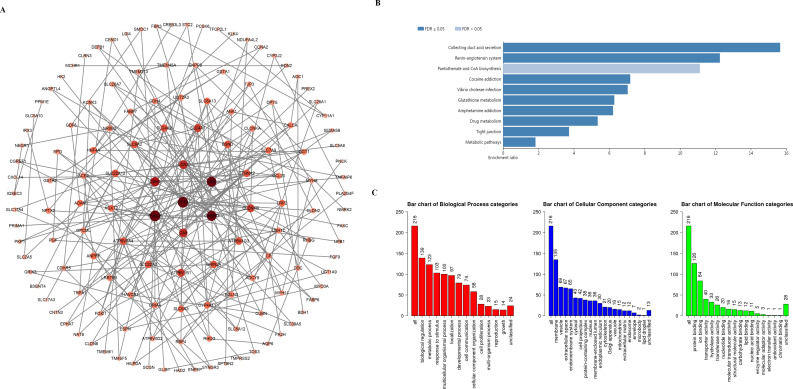
PPI network and the functional enrichment analysis of SIRT family members. (A) STRING and Cytoscape were used to build the PPI network. (B–C) The WebGestalt database was used to analyze the GO functional enrichment and the KEGG pathway of the SIRT-related co-expressed molecules.

To explore the mechanism underlying the functions of SIRTs in ccRCC, we used the cBioPortal and Metascape databases to perform functional analysis. More than 20000 SIRT-correlated genes were downloaded. According to a —log ratio—>1.4 and *P* <0.05, 216 of these were further screened (Table S1). By using the STRING database and Cytoscape, the protein–protein interaction (Fig. 6A) was constructed; Kyoto Encyclopedia of Genes and Genomes (KEGG) enrichment analysis was then conducted by using the WebGestalt database. We found that 10 potential signaling pathways were enriched, including collecting duct acid secretion, renin-angiotensin, pantothenate and CoA biosynthesis, cocaine addiction, *Vibrio cholerae* infection, glutathione metabolism, drug metabolism, amphetamine addiction, tight junction, and metabolic pathways. Among them, the identification of the drug metabolism pathway suggested that the SIRT family has the potential to be a drug target for ccRCC therapy (Fig. 6B). Further, the Metascape and WebGestalt databases were used to analyze Gene Ontology (GO) term enrichment (Fig. 6C). Results shown in Fig. 6C suggested that more than half of the genes were enriched in the membrane. We found that biological regulation was the most enriched biological process category, then was the metabolic process, response to stimulus, and multicellular organismal process.

### Immune cell infiltration related to SIRTs

In recent years, the immune system has been considered a crucial aspect in understanding oncogenesis and cancer progression (Qu et al., 2022). Studies targeting SIRTs have shown that several members of this family are significantly associated with the immune response.

**Figure 7:**
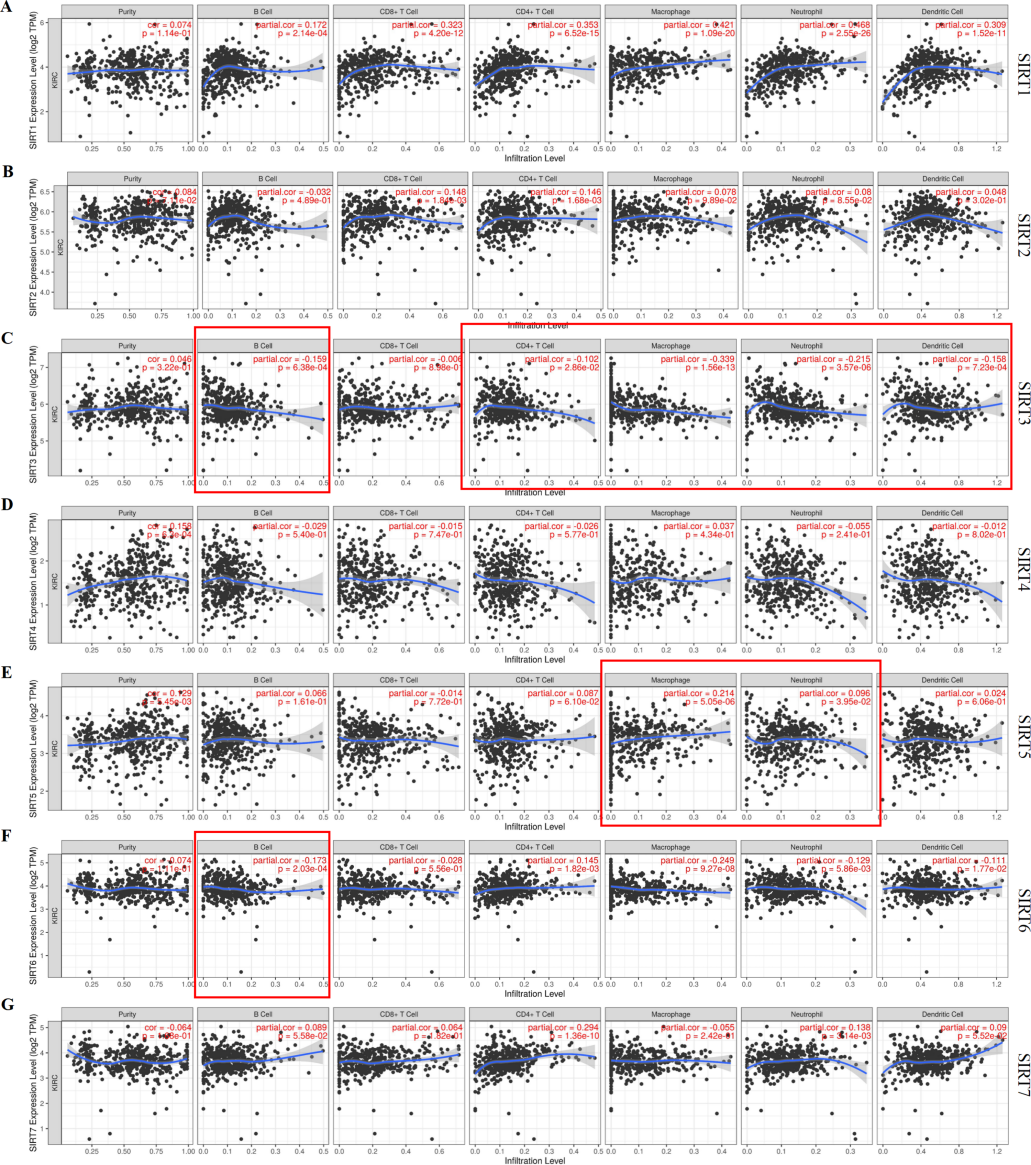
Correlation between tumor-infiltrating immune cells and SIRTs. The data retrieved from the Timer database shows the association of SIRT1 (A) SIRT2 (B) SIRT3 (C) SIRT4 (D), SIRT5 (E) SIRT6 (F) and SIRT7 (G) mRNA expression levels and immune cell infiltration.

Combined with our speculation on the relationship between SIRTs and the immune system, the TIMER database was used to identify any correlations between tumor-infiltrating immune cells and the SIRT family (Figs. 7A–7G). We found that six SIRT family members (all except SIRT4) were related to several common immune cells, including B cells, CD8^+^ T cells, CD4^+^ T cells, macrophages, neutrophils, and dendritic cells. The expression of SIRT1 was positively correlated with all six immune cells. SIRT2 levels were positively correlated with CD8^+^ T cells (Cor = 0.148, *P* = 1.84e−03), and CD4^+^ T cells (Cor = 0.146, *p* =1.68e−03). Figure 7C shows that SIRT3 expression was negatively correlated with B cells (Cor = −0.159, *P* = 6.38e−04), CD4^+^ T cells (Cor = −0.102, *P* = 2.86e−02), macrophages (Cor = −0.339, *P* = 1.56e−13), neutrophils (Cor = −0.215, *P* = 3.57e−06), and dendritic cells (Cor = −0.258, *P* = 7.23e−04). SIRT5 levels were found to be positively correlated with macrophages (Cor = 0.214, *P* = 5.05e−06) and neutrophils (Cor = 0.096, *P* = 3.95e−02), as shown in Fig. 7E. SIRT6 expression was found to be negatively correlated with B cells (Cor = −0.173, *P* = 2.03e−04), macrophages (Cor = −0.249, *P* = 9.27e−08), neutrophils (Cor = −0.129, *P* = 5.86e−03), and dendritic cells (Cor = −0.111, *P* = 1.77e−02) but positively correlated with CD4+ T cells (Cor = 0.145, *P* = 1.82e−03). The expression of SIRT7 was positively correlated with CD4+ T cells (Cor = 0.294, *P* = 1.36e−10) and neutrophils (Cor = 0.138, *P* = 3.14e−03). These data suggest that SIRTs might have an important role in ccRCC through their influence on the infiltration of different immune cells by some certain mechanism. For example, the upregulation of SIRT3 and SIRT6 promoted ccRCC may be mediated by with the increasing immune cell infiltration levels, including B cells, CD4+ T cells, macrophages, neutrophils, and dendritic cells. The downregulation of SIRT5 may be related to the infiltration of macrophages and neutrophils.

## Discussion

SIRTs comprise a type of class III histone deacetylases that depend on NAD (Yu et al., 2019); however, their role in neoplasia is controversial. Some members in this gene family can promote tumorigenesis and development, whereas others are mainly tumor suppressors (Wu et al., 2022). Further, many studies support the irreplaceable role of SIRTs in tumorigenesis. Recently, the incidence of kidney cancer has been increasing. In 2020, 431,288 people were diagnosed with renal cell carcinoma and 179,368 patients died because of this disease (Sung et al., 2021). As the most common form of kidney cancer with a high mortality rate and poor response to treatment, it is necessary to find novel biomarkers for ccRCC diagnosis and treatment. Previous studies found that the overexpression of SIRT4 significantly reduces the proliferation, migration, and invasion ability of ccRCC cells (Wang et al., 2020). Moreover, studies suggest that the expression of SIRT5 is decreased in ccRCC and that it can induce tumorigenesis and progression by accelerating the Warburg effect through PDHA1 hyper-succinylation. This indicates that SIRT5 might be a possible ccRCC suppressor (Yihan et al., 2021). Other members in this family were also proven to be important for the tumorigenesis of many cancers. The results of all of these studies are consistent with our current findings.

In our study, combined with data above from different bioinformatic databases and our experiments (IHC), the role of SIRT5 and other SIRT family members toward the progression and prognosis of ccRCC was verified. We found that the expression levels of SIRT1, SIRT2, SIRT3, SIRT6, and SIRT7 were increased in ccRCC tissues, whereas those of SIRT4 and SIRT5 (particularly SIRT5) were decreased. Our results based on the clinical-pathological characteristics of patients with ccRCC showed that the expression of SIRT2, SIRT3, SIRT6, and SIRT7 was increased in tumor-stage 1-4 subgroups, SIRT1 was increased in tumor-stage 1, and the expression of SIRT4 and SIRT5was decreased. The results of the expression of SIRTs based on different tumor grades showed the same trend. In terms of prognosis, the OS curves suggested that high expression of SIRT4 and SIRT5 was significantly related to longer OS, but high expression of SIRT6 and SIRT7 were shown related to shorter OS. Moreover, the RFS curves suggested that increased expression of SIRT3 was related to shorter RFS, whereas increased SIRT5 was related to longer RFS. Based on the data above, we speculated that SIRT5 may be a potential biomarker of the ccRCC, which inhibits the growth of tumor. Consequently, we did IHC and data analysis based on scoring immunohistochemistry intend to verified it. We found that the expression of SIRT5 was significantly higher in normal adjacent tissues than in ccRCC tissues. And data analysis based on scoring immunohistochemistry showed that aberrant expression of SIRT5 in ccRCC was closely related with pathological grading and cancer size with significant statistically difference (*p* < 0.05) according to Univariate analysis. Further, Multivariate analysis results showed that age (*p* < 0.005), pathological grading (*p* < 0.005), T stage (*p* < 0.005) and SIRT5 expression (*p* < 0.05) were four independent risk factors. Next, we further found that the methylation levels of SIRT4 and SIRT5 were increased, whereas those of SIRT6 and SIRT7, were decreased. Combined with previous studies on SIRT family members in different tumors (Huang et al., 2021), we speculate that the SIRT family have the potential to become new biomarkers and therapeutic targets for ccRCC. Among them, SIRT5 seem to play important roles in tumor suppression. On this basis, we proposed that the functions of these genes require a better and deeper exploration.

Next, we analyzed the mechanism of SIRTs in ccRCC. We used the cBioPortal database to explore the genes correlated with the SIRT family and then retrieved the functional enrichment analysis data from the WebGestalt databases together. These genes were found to be related to some signaling pathways, including the nuclear receptors meta pathway and PID HIF1 TFPATHWAY. PA previous study demonstrated the relationship between the SIRT family members and some important signaling pathways. For example, Ubaid et al. found that SIRT1 could maintain HIF-1α in a deacetylated state to inhibit oxidative stress (Ubaid et al., 2022). Moreover, owing to the important role of immune cells in the tumor microenvironment (the internal environment that is very important for tumorigenesis and development), we studied several important immune cells and the relationship between their signature markers and the expression of SIRTs in ccRCC using the TIMER database. Previous studies targeting SIRTs in immune cells suggested that SIRT family members are significantly associated with the immune response. For example, by studying the potential roles of SIRTs in different subtypes of T cells during the adaptive immune response, Hamaidi et al. speculated that SIRTs are of significant interest as therapeutic targets to treat immune-related diseases and enhance antitumor immunity (Hamaidi & Kim, 2022). SIRT3 was suggested by Huang et al. (2021) as a crucial marker which has a marked influence on immune cell functions. Also, SIRT6 was further found to be capable of delaying the onset of experimental autoimmune encephalomyelitis (Piacente et al., 2022). Combining these results, we conjecture that the expression of some of the SIRT family members are associated with various immune cells in ccRCC and most of their marker genes.

## Conclusions

In conclusion, the molecular profiles of the SIRT family were retrieved from several bioinformatic databases. Based on experiments (IHC) and data analysis, we speculated that the expression of SIRTs may be important for ccRCC, among them, SIRT4 and SIRT5 (particularly) could be involved in inhibiting the progression of ccRCC, whereas SIRT6 and SIRT7 are speculated to be important for promoting ccRCC development. We think these findings may be important for further understanding the mechanism of the tumorigenesis of ccRCC, and could provide more details to predict the prognosis of patients with ccRCC and identify more new treatment strategies.

##  Supplemental Information

10.7717/peerj.15154/supp-1 Supplemental Information 1 Raw Data

10.7717/peerj.15154/supp-2 Supplemental Information 2 A —log ratio—< 1.4 and *P*< 0.05, 216 of these were further screened

## Abbreviations

SIRTs: sirtuins
NAD^+^: nicotinamide adenine dinucleotide
ccRCC: clear cell renal cell carcinoma
HCC: hepatocellular carcinoma
IHC: immunohistochemistry
GEPIA: gene expression profiling interactive analysis
THPA: The Human Protein Atlas
ANOVA: analysis of variance
IHC: immunohistochemistry
TIMER: tumor immune estimation resource
FP: first progression
OS: overall survival
RFS: recurrence free survival
KEGG: Encyclopedia of Genes and Genomes
GO: Gene Ontology

## Additional Information and Declarations

### Data Availability

The following information was supplied regarding data availability: The raw data is available in the Supplemental Files.
